# Cu-TCPP Nanosheets-Sensitized Electrode for Simultaneous Determination of Hydroquinone and Catechol

**DOI:** 10.3390/ma15134625

**Published:** 2022-06-30

**Authors:** Liudi Ji, Qi Wang, Lianhui Peng, Xiaoyu Li, Xiaoming Zhu, Peng Hu

**Affiliations:** 1 Hubei Key Laboratory of Radiation Chemistry and Functional Materials, School of Nuclear Technology and Chemistry & Biology, Hubei University of Science and Technology, Xianning 437100, China; jiliudi@126.com (L.J.); hy33601@163.com (Q.W.); h15038177813@163.com (L.P.); 2 School of Electronic and Electrical Engineering, Hubei Province Engineering Research Center for Intelligent Micro-Nano Medical Equipment and Key Technologies, Wuhan Textile University, Wuhan 430200, China

**Keywords:** hydroquinone, catechol, electrochemical sensor, Cu-TCPP, 2D MOFs

## Abstract

It is quite important to develop sensitive, simple, and convenient methods for the simultaneous determination of Hydroquinone (HQ) and Catechol (CC) due to their wide existence, the difficulty of degradation, and the high toxicity. Herein, Cu-TCPP nanosheets were prepared in *N*,*N*-dimethylformamide (DMF) through the solvent exfoliation method. The morphology and electrochemical performance of Cu-TCPP were characterized, revealing its stacked sheet structure with abundant pores, a fast electron transfer ability, and a large electrode active area. Using Cu-TCPP nanosheets as the sensitive material to modify the glassy carbon electrodes (Cu-TCPP/GCEs), it was found that they had an obvious enhancement effect on the electrochemical oxidation currents of HQ and CC. The signal enhancement mechanism was explored. The Cu-TCPP nanosheets not only enhanced the accumulation abilities of HQ and CC, but also improved their apparent catalytic rate, displaying high sensitivity for HQ and CC. The values of the detection limit were calculated to be 3.4 and 2.3 nM for HQ and CC. A satisfactory recovery was obtained when this method was used in measuring HQ and CC in the water samples.

## 1. Introduction

Hydroquinone (HQ) and Catechol (CC) are two main isomers of benzenediol compounds, widely used in the production of cosmetics, medicines, textiles, and pesticides. They often coexist in the wastewater of these factories. On account of its high toxicity and the difficulty in degradation [1,2], it is essential to develop a rapid, sensitive, and accurate environmental analysis method for the determination of HQ and CC. Many methods have been established for their simultaneous determination, including chromatography [3,4], spectrophotometry [5], chemiluminescence [6], and fluorescence [7,8]. In addition, due to the advantages of low price, easy operation, quick response, and high sensitivity, the electrochemical method has attracted more attention. A variety of modified electrodes have been constructed [9,10,11,12,13,14,15]. However, the simplicity and sensitivity of these reported electrochemical methods still need to be improved.

Developing new electrochemical sensitive materials is crucial for the exposition of highly sensitive and selective electrochemical sensing mechanisms and the construction of high-performance electrochemical sensors. Metal–organic frameworks (MOFs), a special class of multifunctional porous material, are assembled by the coordination of metal ions or clusters with organic ligands. MOFs have good applications in electrochemical sensing because of their large surface area, ultrahigh porosity, and unsaturated metal sites [16,17]. Among that, the Cu-MOFs are widely used due to the excellent redox activity of Cu^2+^. For example, Cu-BTB (BTB: benzene-1,3,5-tribenzoate), N-Cu-BTC (BTC: 1,3,5-Benzenetricarboxylic acid), Cu-BTC, and Cu-BDC (BDC: 1,4-Benzenedicarboxylic acid) have been successfully used in developing the electrochemical sensors for nitrite, dopamine, sulfanilamide, malachite green, and estradiol [18,19,20,21]. However, at present, most of the MOFs used are 3D bulk materials with large sizes or thicknesses, and the performance has not been fully achieved [18,22]. 2D MOF nanosheets are new members of 2D materials and possess an ultrathin thickness morphology and more accessible active sites, allowing for an ultrahigh surface area, rapid mass transport, superior electron transfer, and high catalytic activity [23,24]. The electrochemical sensing efficiency will effectively improve.

Having said all of above, this study aims to evaluate a novel electrochemical method for the simultaneous determination of HQ and CC based on the Cu-TCPP nanosheets. The Cu-TCPP nanosheets provide a faster electron transfer ability, larger surface area, better adsorption ability, and higher electrocatalytic rate for the oxidation of HQ and CC. Finally, using Cu-TCPP nanosheets as sensing material, a simple and highly sensitive sensor has been constructed for HQ and CC. The method was successfully applied in lake water samples with a satisfactory recovery. Simple materials were used and good results were achieved. This work will open a new thought for the rapid detection of trace pollutants in complex water environment samples. It is helpful to construct superior electrochemical sensing systems.

## 2. Materials and Methods

### 2.1. Reagents and Solutions

Copper(II) nitrate trihydrate (Cu(NO_3_)_2_·3H_2_O) and DMF were bought in Sinopharm Chemical Reagent Co., Ltd. (Shanghai, China). Tetrakis(4-carboxyphenyl)porphyrin (H_2_TCPP), Hydroquinone (HQ), and Catechol (CC) were purchased from Aladdin Chemistry Co., Ltd. (Shanghai, China). All chemicals were used without further purification.

### 2.2. Instruments

The X-ray diffraction (XRD, X’Pert PRO diffractometer, Cu Ka, Panalytical Company, Almelo, The Netherlands) was employed to characterize the crystal structure. A Fourier transform infrared (FT-IR) was analyzed on an Avatar 360 Nicolet instrument (Thermo Fisher Scientific, Shanghai, China). Scanning electron microscopic images and transmission electron microscopic images were measured using a Nova Nano SEM 450 system and the Tecnai G2 F30 system, respectively (FEI Company, Eindhoven, The Netherlands). A micromeritics ASAP 2020 analyzer (Norcross, GA, USA) was used to test Nitrogen adsorption–desorption isotherms.

A CHI 660E electrochemical workstation (Shanghai Chenhua Instrument Co., Ltd., Shanghai, China) was used for electrochemical measurements. The Cu-TCPP modified glassy carbon electrode (GCE, diameter: 3 mm), saturated calomel electrode (SCE), and platinum wire were used as the working electrode, reference electrode, and counter electrode, respectively.

### 2.3. Construction of Cu-TCPP Nanosheets Modified Electrode

In a typical process, 0.2174 g of Cu(NO_3_)_2_·3H_2_O and 0.2372 g of H_2_TCPP were dissolved in 45.0 mL of DMF and 15.0 mL of ethanol. Then, the above solution was heated in an oven at 80 °C for 24 h. The resulting precipitates were centrifuged, washed with ethanol three times, and then dried in a vacuum chamber at 60 °C for 12 h. After that, 6.0 mg of the samples were dispersed into 6.0 mL of DMF with the aid of ultrasonication (2 h). After centrifuging (2000 rpm, 20 min), the supernatant was collected as Cu-TCPP nanosheets suspension.

For the modification, the GCE was first polished with 0.05 μm of Al_2_O_3_ slurry and was then ultrasonically washed in ethanol and ultrapure water. Then, 3.0 μL of Cu-TCPP nanosheets suspension was coated on the GCE to prepare a Cu-TCPP nanosheets modified GCE (Cu-TCPP/GCE).

## 3. Results

### 3.1. Characterization of Cu-TCPP Nanosheets

**Figure 1:**
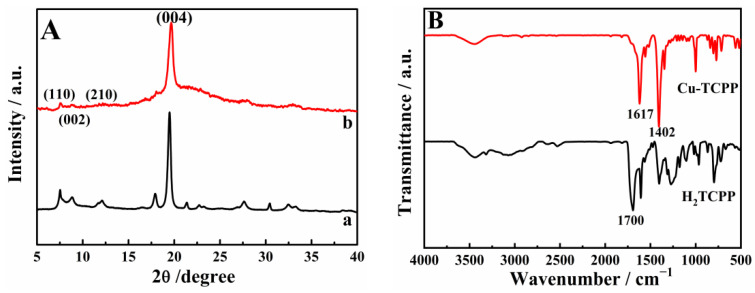
(**A**) XRD pattern of the obtained Cu-TCPP by the reported method (**A**) and this work (**B**); (**B**) FTIR spectra of H_2_TCPP and the prepared Cu-TCPP nanosheets.

The XRD pattern of the Cu-TCPP nanosheets was recorded in Figure 1A. Four typical peaks at 7.8°, 8.8°, 12.2°, and 19.6° were found, which were corresponding to the featured (110), (002), (210), and (004) plane. The phenomenon was in good agreement with the results of Cu-TCPP prepared by the reported method [25], indicating the successful preparation of Cu-TCPP. Then, FTIR was used to analyze the binding mode of the Cu-TCPP nanosheets. As shown in Figure 1B, a strong peak at approximately 1700 cm^−1^ in the FTIR spectrum of H_2_TCPP was observed, which was corresponding to the C=O stretching band. However, the peak became almost invisible in Cu-TCPP, and two new peaks exhibited near 1617 and 1402 cm^−1^ represented the OC-O-Cu bond [25,26,27]. This confirms the formation of coordination bonds between Cu^2+^ and the carboxyl groups of Cu-TCPP.

**Figure 2:**
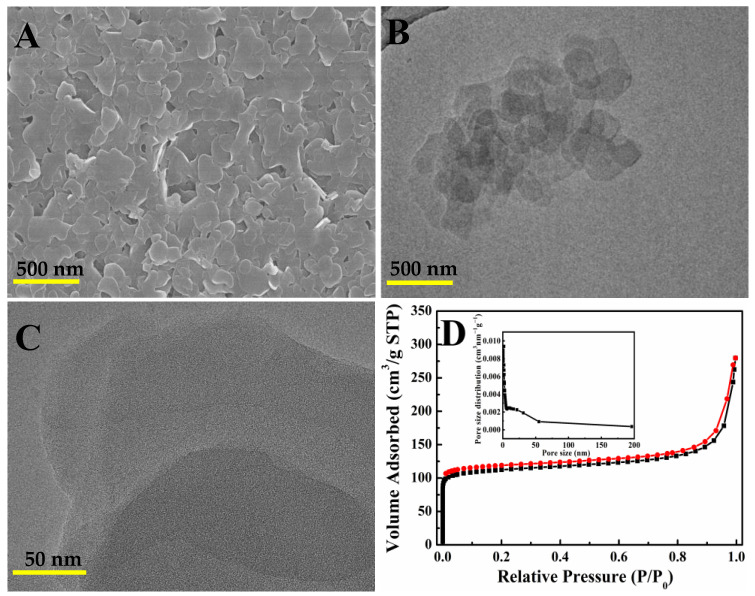
SEM (**A**) and TEM images (**B**,**C**) of Cu-TCPP nanosheets; (**D**) Nitrogen sorption isotherms and pore size distribution (inset) of Cu-TCPP.

SEM and TEM were used to investigate the morphological features of the synthetic Cu-TCPP nanosheets systematically. Firstly, the morphology of the original bulk Cu-TCPP was characterized. As shown in Figure S1, the bulk Cu-TCPP MOFs had a closely packed multilayer structure. After the ultrasonic exfoliation, a sheet-like morphology was observed, and the degree of stacking between the layers was significantly reduced (Figure 2A,B). It is interesting to note that many pores were observed on the nanosheets (Figure 2C). Furthermore, as given in Figure 2D, N_2_ adsorption–desorption experiments were applied to further characterize the porous structures of the as-prepared Cu-TCPP samples. The average pore diameter evaluated by the Barrett–Joyner–Halenda (BJH) equation on the desorption branching was 2.12 nm, and the total pore volume was 0.43 cm^3^/g. The specific surface area was calculated to be 410.4 m^2^/g according to the Brunauer–Emmett–Teller (BET) equation. The result was comparable to its reported values of 391.2 m^2^ g^−1^, 478 m^2^ g^−1^, and 485 m^2^ g^−1^ [28,29,30]. Undoubtedly, the abundant porous structure and high specific surface area would endow large electrochemical active areas and many active sites.

**Figure 3:**
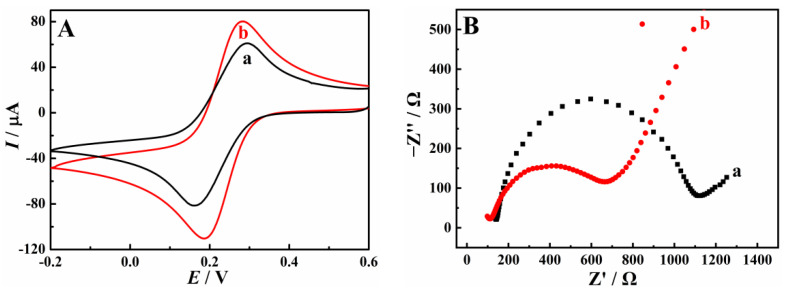
(**A**) CV curves of 5.0 mM of K_3_[Fe(CN)_6_] (1.0 M KCl) on GCE (a) and Cu-TCPP/GCE (b); (**B**) Nyquist impedance plots of GCE (a) and Cu-TCPP/GCE (b) in 0.1 M KCl containing 5 mM of K_3_/K_4_Fe(CN)_6_, frequency range: 100 kHz to 0.1 Hz; amplitude: 5 mV.

Employing K_3_[Fe(CN)_6_] as the redox probe, the electrochemical properties of the Cu-TCPP nanosheets were investigated. As Figure 3A shows, a pair of redox peaks was observed on both GCEs and the signals increased on the surface of the Cu-TCPP/GCE, indicating an enlarged active area. Furthermore, it is worth noting that the oxidation peak shifted to the left and the reduction wave shifted to the right on the surface of Cu-TCPP/CPE, suggesting the enhanced electron transfer ability. Electrochemical impedance spectroscopy (EIS) was applied to further confirm the electron-transfer abilities of the Cu-TCPP nanosheets. As shown in Figure 3B, a large semicircle in good shape was found on the GCE (curve a) and the semicircle decreased on the surface of the Cu-TCPP/GCE (curve b). According to the Randles equivalent circuit, the values of the charge transfer resistance (*R*_ct_) were fitted to be 963.5 and 648.9 Ω on the GCE and Cu-TCPP/GCE. The smaller *R*_ct_ indicates that the porous Cu-TCPP nanosheets facilitated the electron transfer.

**Figure 4:**
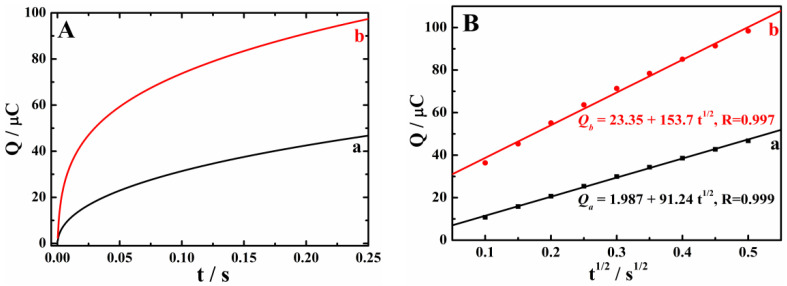
*Q*-*t* curves (**A**) and *Q*-*t*^1/2^ plots (**B**) of 5.0 mM of K_3_[Fe(CN)_6_] in 1.0 M KCl on GCE (a) and Cu-TCPP/GCE (b).

Then, the chronocoulometry method was used to further check the electrochemically active area of the bare GCE and Cu-TCPP/GCE. As presented in Figure 4A,B, the curves of charge (*Q*)-time (*t*) are given and then converted to *Q*-*t*^1/2^ straight lines. Based on the Cottrell equation [31]:

*Q* = 2nFA*cD*^1/2^ π^−1/2^
*t*^1/2^ + *Q*_dl_ + *Q*_ads_ (1)

the electrochemically active area (A) was easily calculated to be 0.061 and 0.102 cm^2^ for the GCE and Cu-TCPP/GCE through the slope of the linear equation. The enlarged electrochemically active area probably originated from the porous sheet-like structure, which further provided more catalytic active sites and accumulation efficiency towards targets.

### 3.2. Enhanced Oxidation of HQ and CC on Cu-TCPP/GCE

**Figure 5:**
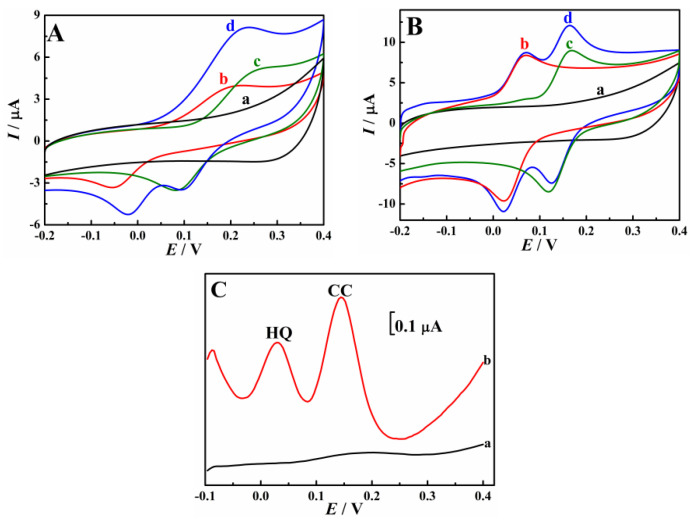
CV behaviors of GCE (**A**) and Cu-TCPP/GCE (**B**) in pH 7.0 PBS: (a) blank solution; (b) with 100 μM of HQ; (c) with 100 μM of CC; (d) with 100 μM of HQ and CC, scan rate of 100 mV s^−1^; (**C**) DPV curves of 2 µM of HQ and CC on GCE (a) and Cu-TCPP/GCE (b) in pH 7.0 PBS.

The CV behaviors of HQ and CC on different GCEs were analyzed (Figure 5A,B). Curve a represents the CV response in 0.1 M phosphate buffer solution (pH = 7.0); no peak was observed. After 100 µM of HQ or CC was added, the response is shown as curves b and c, and a pair of redox peaks was noticed. When 100 μM of HQ and CC were added at the same time, the CV response is given as curve d. One overlapping oxidation peak was found on the GCE, indicating poor sensitivity and selectivity for the simultaneous determination of HQ and CC. However, there were still two peaks on the surface of the Cu-TCPP/GCE, which were consistent with the signals of the single addition. By comparing the GCE and Cu-TCPP/GCE, it can be seen that the oxidation peaks of HQ and CC on the surface of the Cu-TCPP/GCE had a good shape and degree of separation. This was mainly due to the larger specific surface area, porous structure, and faster electron transfer ability of Cu-TCPP, which was good for the selective accumulation and oxidation of HQ and CC. Moreover, the peak currents increased, and the peak potentials shifted negatively, indicating that Cu-TCPP showed a high catalytic ability toward the oxidation of HQ and CC. As previously reported, Cu^2+^ can be seen as the catalytic activity center [32,33]. In conclusion, the Cu-TCPP nanosheets can be used as sensitization material for the simultaneous determination of HQ and CC. The CV curves of HQ and CC with different concentrations on the Cu-TCPP/GCE were studied, and the results are shown in Figure S2A. The oxidation peak enhanced linearly with concentrations over the range from 1 to 200 μM. The linear correlation equation and the correlation coefficient are given in Figure S2B,C, indicating a good linear relationship.

Then, the voltammetric determination of 2.0 μM of HQ and CC was studied using DPV. The response of HQ and CC on different GCEs is compared in Figure 5C. A little bump was found on the GCE (curve a). After the modification of the Cu-TCPP nanosheets on the GCE, two obvious peaks were presented, the oxidation peak currents obviously increased, and the oxidation waves shifted negatively. The significantly enhanced oxidation currents and the left shift of the oxidation potentials indicated that the prepared Cu-TCPP nanosheets exhibited excellent electrochemical reactivity toward the oxidation of HQ and CC.

**Figure 6:**
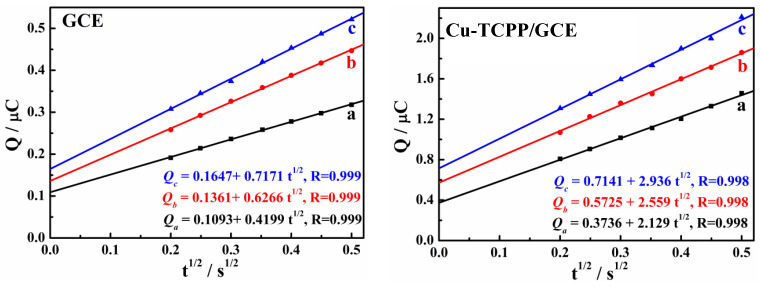
*Q*-*t*^1/2^ plots on different GCEs in pH 7.0 phosphate buffer (curve a) and in the presence of 2 µM of HQ (curve b) or 2 µM of CC (curve c).

The adsorption behaviors of HQ and CC on different GCEs were studied using chronocoulometry to discuss the origin of the signal enhancement effects of the Cu-TCPP/GCE. The *Q*-*t*^1/2^ straight lines of the GCE (A) and Cu-TCPP/GCE (B) shown in Figure 6 were transferred from the recorded *Q*-*t* curves. Based on the integrated Cottrell equation (Equation (1)), *Q*_dl_ is the intercept of the *Q*-*t*^1/2^ plot in blank pH 7.0 PBS (curve a) while the intercept value in the presence of HQ or CC (curve b or c) represents the summation of *Q*_dl_ and *Q*_ads_. Therefore, the *Q*_ads_ of HQ and CC on the GCE and Cu-TCPP/GCE are easily calculated from Figure 6. The results are given in Table 1. The bigger *Q*_ads_ on the surface of Cu-TCPP indicates the increased accumulation abilities, and led to the enhanced signals.

**Figure 7:**
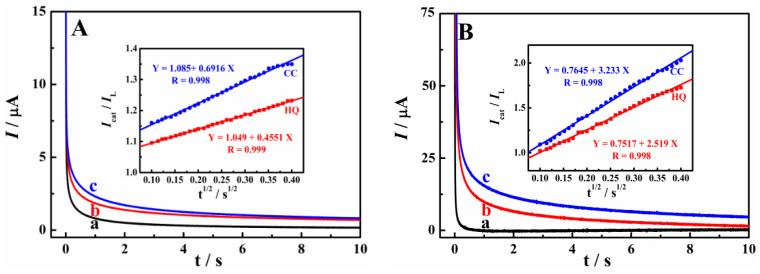
Chronoamperometry behaviors on GCE (**A**) and Cu-TCPP/GCE (**B**) in the absence (a) or presence of 1 mM of HQ (b), 1 mM of CC (c) in pH 7.0 PBS, inset: plots of *I*_cat_/*I*_L_-*t*^1/2^.

Then, in order to further understand the significantly increased response signals, the chronoamperometry experiment was used to explore the electrochemical kinetics. Figure 7 gives the *I*-*t* plot of the GCE (A) and Cu-TCPP/GCE (B) in 0.1 M 7.0 PBS (a) or in the presence of 1 mM of HQ (b) or 1 mM of CC (c). According to the bottom equation, the apparent catalytic rate constant (*k*_cat_) can be calculated [27]:

*I*_cat_/*I*_L_ = (π*k*_cat_*C_o_t*)^1/2^ (2)

where *I*_cat_ is the catalytic current in the presence of analytes and *I*_L_ is the limiting current in the absence of analytes. The linear relationship between *I*_cat_/*I*_L_ and *t*^1/2^ is plotted in the inset of Figure 7B. The *k*_cat_ on the Cu-TCPP/GCE was calculated to be 1.527 × 10^3^ for HQ and 3.327 × 10^3^ for CC, which is larger than those of the GCE (65.92 for HQ and 152.3 for CC). The results indicate that the electro-oxidation of HQ and CC on the surface of the Cu-TCPP/GCE showed a higher electrocatalytic rate. The possible reasons are summarized as follows: (1) the large specific surface area and abundant porous structure were beneficial to the efficient accumulation of HQ and CC; (2) the porous structure could facilitate the electron transfer of the electrochemical oxidation of HQ and CC; (3) the Cu^2+^ in Cu-TCPP nanosheets could be seen as the catalytic activity center, having a high catalytic ability toward the oxidation of HQ and CC.

### 3.3. Electrochemical Reaction Mechanism of HQ and CC on Cu-TCPP/GCE

**Figure 8:**
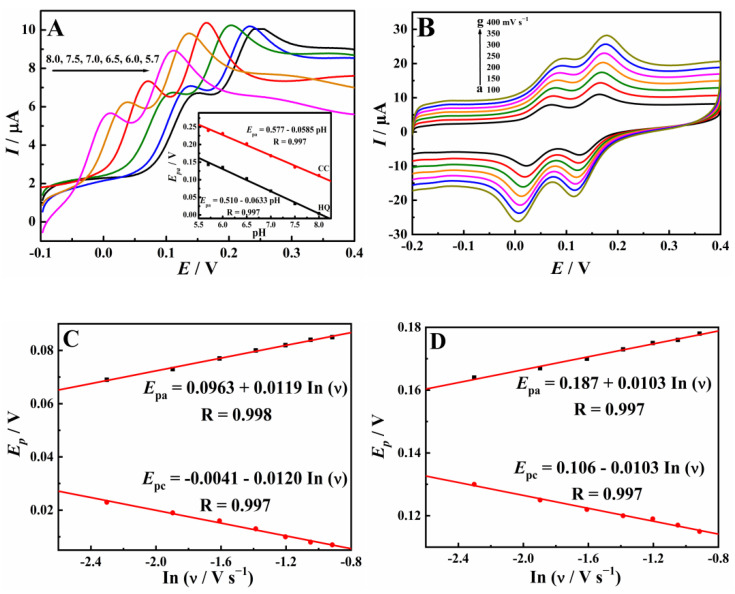
(**A**) LSV curves of 100 μM of HQ and CC on Cu-TCPP/GCE in phosphate buffers with different pH values, inset: *E*_pa_-pH plot; (**B**) CV curves of 100 μM of HQ and CC on Cu-TCPP/GCE in pH 7.0 PBS with different scan rate; (**C**,**D**) plots of *E*_p_–ln (ν).

As given in Figure 8A, in different buffer solutions, the oxidation behaviors of HQ and CC on the Cu-TCPP/GCE were compared using linear sweep voltammetry (LSV). The relationship between the different pH values and oxidation peak potentials (*E*_pa_) was studied. As shown in the inset of Figure 8A, both *E*_pa_ and pH had a good linear relationship. The slope values were 63.3 mV pH^−1^ and 58.5 mV pH^−1^, suggesting that the involved electrons and protons in the process of HQ and CC oxidation were equal in number. Moreover, with the pH value increased from 5.8 to 7.0, the oxidation peak currents (*I_pa_*) on the Cu-TCPP/GCE gradually increased and then steadily decreased. Therefore, pH 7.0 PBS was used for the subsequent studies.

**Scheme 1:**
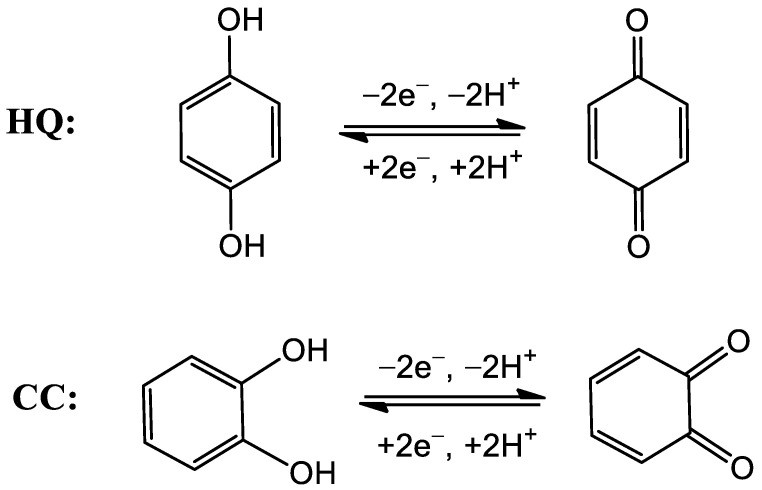
The oxidation mechanism for HQ and CC.

In order to further explore the reaction mechanism of HQ and CC on the Cu-TCPP/GCE, their electrochemical behaviors were studied using CV under different scan rates (from 100 to 400 mV s^−1^). As shown in Figure 8B, two pairs of redox waves were found, and the *I*_p_ linearly increased with the square root of the scan rates, suggesting a diffusion-controlled electrode process. Furthermore, *E*_pa_ moved positively and *E*_pc_ moved negatively with the scan rate increasing. As given in Figure 8C,D, the plot of *E*_p_-ln(ν) was a straight line. Thus, according to the Laviron theory [31], the αn value was obtained to be 1.07 and 1.24 for HQ and CC. Here, α was considered as 0.5, so the value of n was two. Therefore, as shown in Scheme 1, the oxidation of HQ and CC was both a two-proton and two-electron process. which is in good agreement with the reported results [8].

### 3.4. Simultaneous Determination of HQ and CC

**Figure 9:**
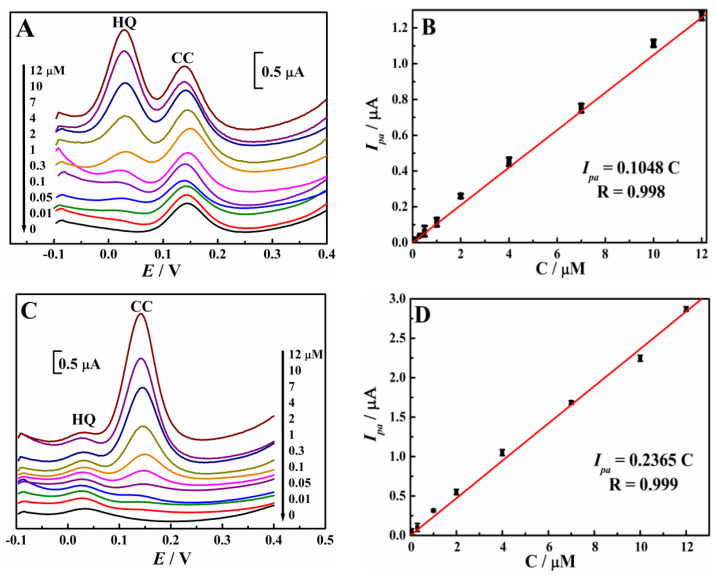
(**A**) DPV behaviors of 2 µM of CC with varying concentrations of HQ. (**B**) Calibration plots for HQ. (**C**) DPV behaviors of 2 µM of HQ with varying concentrations of CC. (**D**) Calibration plots for CC. The electrode is a Cu-TCPP /GCE. Error bar represents the standard deviation of triple measurements.

The analytical performance of a Cu-TCPP/GCE toward the simultaneous determination of HQ and CC was explored using DPV. The DPV curves with different concentrations of HQ and CC were recorded, and the *I*_pa_ moved linearly with their concentrations (*C*) (Figure 9); the linear regression equations are given in Figure 9B,D. As a result, the linear range and detection limit (based on three signal-to-noise ratios) was 0.01–12 µM and 3.4 nM for HQ, and 0.01–12 µM and 2.3 nM for CC. In comparison with the other systems (Table 2), the Cu-TCPP/GCE exhibited lower detection limits.

The reproducibility of the fabricated electrochemical platform of the Cu-TCPP/GCE was studied. The relative standard deviations (RSD) of the twelve tests were 3.5% (HQ) and 5.3% (CC), suggesting high reproducibility. The interference of some inorganic ions and possibly coexisting organic compounds on the simultaneous determination of 2 µM of HQ and CC was examined. A 2000-fold concentration of NO_3_^−^, Cl^−^, SO_4_^2−^, Na^+^, K^+^, and Ca^2+^, a 100-fold concentration of 4-chlorophenol, p-nitrophenol, and bisphenol A, and a 30-fold concentration of resorcin and phenol were added respectively, and no noticeable change (less than 5%) was found in the detected signals. The above results indicate that the Cu-TCPP-DMF/GCE had outstanding reproducibility and selectivity for the simultaneous determination of HQ and CC.

Furthermore, to evaluate the feasibility of a Cu-TCPP/GCE, the newly developed sensing platform was applied to analyze local lake water samples by spike and recovery experiments. Before testing, the samples were filtered and diluted one time using pH 7.0 PBS. Each sample was measured in parallel three times and the RSDs for these measurements were below 5.0%. A recovery range between 95.72% and 106.1% is shown in Table 3, indicating that the Cu-TCPP/GCE can be used for practical applications accurately and reliably.

## 4. Conclusions

A sensitive and simple electrochemical sensor was constructed for the simultaneous determination of HQ and CC by using a Cu-TCPP nanosheets modified electrode. The Cu-TCPP nanosheets showed a signal enhancement effect toward HQ and CC. It was derived from its increased electron transfer ability, electrode active area, accumulation ability, and apparent catalytic rate. The newly developed method had low detection limits, good reproducibility, and excellent selectivity. Moreover, the sensor was successfully applied to analyze HQ and CC in lake water samples, indicating good sensing application prospects.

## Figures and Tables

**Table 1:** Comparison of *Q*_ads_ for HQ and CC on the GCE and Cu-TCPP/GCE.

Electrode	*Q*_ads_ (µC)	
	HQ	CC
GCE	0.0268	0.0554
Cu-TCPP/GCE	0.1989	0.3405

**Table 2:** Comparison of different methods for the determination of HQ and CC.

Modified Electrode	Linear Range (µM)		LOD (nM)		Ref.
	HQ	CC	HQ	CC	
Co_3_O_4_@C/GNPs/GCE	0.04–30	0.5–30	14.7	169	[7]
OV-LDHs/H-MWCNTs/GCE	0.5–150	0.5–150	76	74	[8]
BNC/GCE	0.099–8400, 8400–43,340	0.049–1750, 1750–5110	33.3	16.3	[9]
MCPBAC/GCE	0.6–100, 100–600	0.6–100, 100–600	200	200	[10]
WBC/Au/GCE	0.008–1.0, 1.0–7.0	0.01–1.0, 1.0–7.0	2.0	4.0	[12]
AuNPs-MPS/CPE	10.0–1000.0	30.0–1000.0	1200	1100	[13]
CS/MWCNTs/PDA/AuNPs/GCE	0.1–10	0.1–10	35	47	[14]
Cu-TCPP/GCE	0.01–12	0.01–12	3.4	2.3	This work

**Table 3:** Determination of HQ and CC in lake water samples.

Samples	Added (nM)		Found (nM)		Recovery (%)	
	HQ	CC	HQ	CC	HQ	CC
A	50.00	50.00	47.92	48.12	95.84	96.24
	250.0	250.0	258.7	263.2	103.5	105.3
	500.0	500.0	486.3	478.6	97.26	95.72
B	1000	1000	1045	1032	104.5	103.2
	3000	3000	2894	2963	96.47	98.77
	5000	5000	5227	5304	104.5	106.1

## Data Availability

Data are contained within the article.

## Supplementary Materials

The following supporting information can be downloaded at: https://www.mdpi.com/article/10.3390/ma15134625/s1, Figure S1: SEM image of the original bulk Cu-TCPP; Figure S2: (A) CV behaviors of HQ and CC with different concentrations on Cu-TCPP/GCE. (a) 1, (b) 5, (c) 10, (d) 50, (e) 100, and (f) 200 µM; (B) Calibration plots for HQ; (C) Calibration plots for CC.
