# Supplementary material for: Cu-TCPP Nanosheets-Sensitized Electrode for Simultaneous Determination of Hydroquinone and Catechol
Source: Materials (Basel). 2022 Jun 30;15(13):4625. doi: 10.3390/ma15134625 (PMC9267553; doi:10.3390/ma15134625)
Supplement: Supplementary file 1 [file materials-15-04625-s001.zip › materials-1777682-supplementary.pdf]

Cu-TCPP Nanosheets-Sensitized Electrode for Simultaneous Determination of Hydroquinone and Catechol

Liudi Ji [1], Qi Wang [1], Lianhui Peng [1], Xiaoyu Li [2,*], Xiaoming Zhu [1,*] and Peng Hu [1,*]

[1] Hubei Key Laboratory of Radiation Chemistry and Functional Materials,

School of Nuclear Technology and Chemistry & Biology, Hubei University of Science and Technology, Xianning 437100, China; jiliudi@126.com (L.J.); hy33601@163.com (Q.W.); h15038177813@163.com (L.P.)

[2] School of Electronic and Electrical Engineering, Hubei Province Engineering Research Center for Intelligent Micro-nano Medical Equipment and Key Technologies, Wuhan Textile University, Wuhan 430200, China

* Correspondence: hupeng@hbust.edu.cn (P.H.), xyli@wtu.edu.cn (X.L.), zhuxiaoming@hbust.edu.cn (X.Z.)

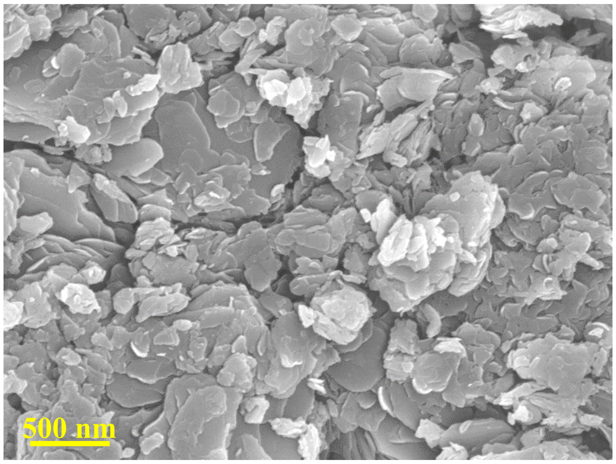

Figure S1. SEM image of the original bulk Cu-TCPP.

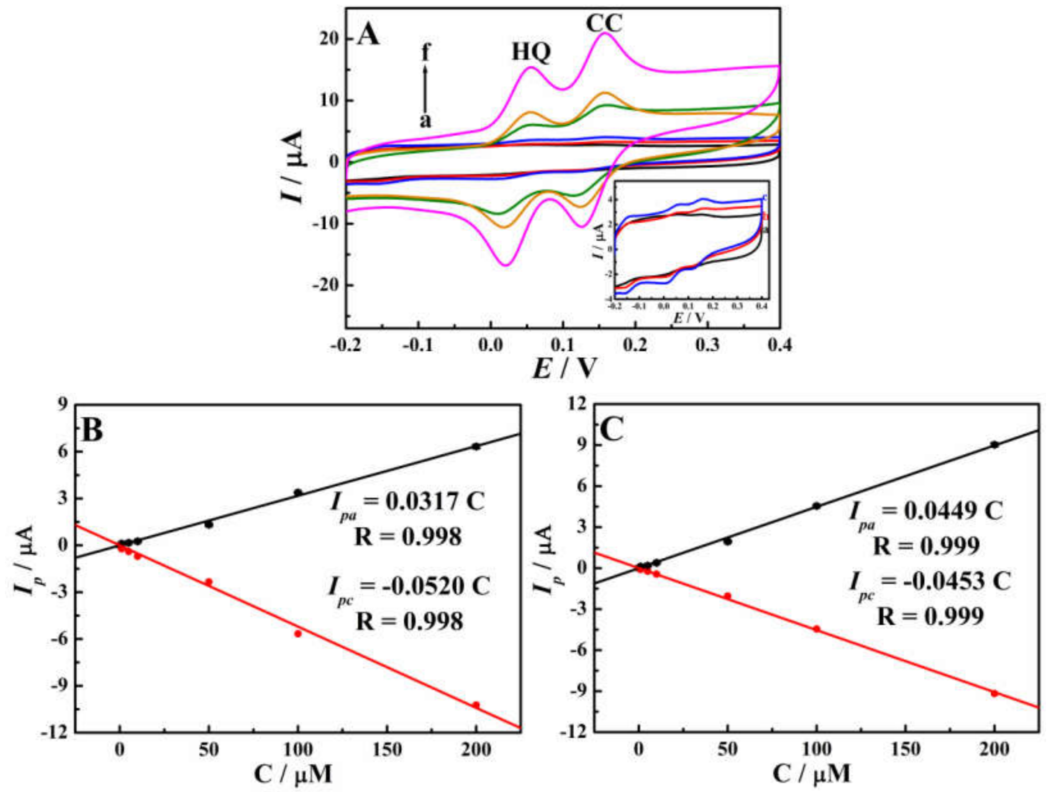

Figure S2. (A) CV behaviors of HQ and CC with different concentrations on Cu-TCPP/GCE. (a) 1, (b) 5, (c) 10, (d) 50, (e) 100, and (f) 200 μM; (B) Calibration plots for HQ; (C) Calibration plots for CC.

.
